# Non-invasive assessment prior to invasive coronary angiography in routine clinical practice in Switzerland – Is it according to the guidelines?

**DOI:** 10.1371/journal.pone.0222137

**Published:** 2019-09-06

**Authors:** Rebecca Schiefer, Hans Rickli, Evelyne Neurauter, Marc Buser, Daniel Weilenmann, Micha T. Maeder

**Affiliations:** Cardiology Department, Kantonsspital St. Gallen, Switzerland; Rutgers Robert Wood Johnson Medical School, UNITED STATES

## Abstract

**Background:**

Non-invasive testing is recommended as a basis to decide about the indication for invasive coronary angiography (ICA) in patients with suspected stenotic coronary artery disease (CAD). However, a recent study based on insurance claims data reported that one third of patients undergoing ICA in Switzerland did not have non-invasive testing beforehand. We aimed to re-evaluate the practice of testing prior to ICA in Switzerland by manual review of patient histories.

**Methods:**

Retrospective analysis of all 816 consecutive patients (age 67±9 years, 70% males) undergoing elective ICA solely for the evaluation of stenotic CAD during the year 2015 in a single center in Eastern Switzerland. The proportion of patients undergoing a non-invasive test was assessed, and predictors of the lack of such a test were determined.

**Results:**

764/816 (94%) patients had a non-invasive test prior to ICA. The majority of patients (728/816; 89%) had an exercise stress test, one fifth (160/816; 20%) underwent a test other than an exercise stress test (6% scintigraphy, 4% stress echocardiography, 6% stress magnetic resonance imaging, 4% computed tomography coronary angiography), and 122/816 (15%) patients had two tests. The use of antianginal drugs other than beta-blockers [odds ratio 1.92 (95% confidence interval 1.01–3.66); p = 0.047] and a lower left ventricular ejection fraction [odds ratio 0.97 (95% confidence interval 0.94–0.99) per one % point increase; p = 0.005] were independent predictors of the lack of a non-invasive test. ICA revealed stenotic CAD in 72% of patients, and 54% of patients underwent revascularization. Patients with and without non-invasive tests did not differ with respect to ICA findings and management.

**Conclusions:**

The present analysis suggests that patients are appropriately selected for ICA based on clinical judgement and non-invasive testing in Switzerland. There is no evidence for an overuse of ICA.

## Introduction

Invasive coronary angiography (ICA) is the current gold standard for the anatomic diagnosis of coronary artery disease (CAD). However, ICA is an invasive procedure associated with a numerically small but existing risk of potentially life-threatening and/or disabling complications including access site bleeding, arrhythmia, stroke, and renal failure [1–3]. Therefore, careful selection of patients for ICA is essential. In particular, ICA should be avoided in subjects with a low likelihood of significant CAD in whom the disease can be excluded by history and other tests [4]. Current guidelines suggest a Bayesian approach of using non-invasive tests depending on a patient’s pre-test probability (PTP) of stenotic CAD as basis to decide whether or not ICA is required [4]. In subjects with a low (<15%) or high (>85%) PTP, tests are not required for purely *diagnostic* purposes. In those with high PTP (>85%), non-invasive tests are used for *risk stratification*. In subjects with an intermediate probability (15–85%), a non-invasive test should be performed to confirm or exclude CAD and as a basis to decide whether or not ICA should be performed. The selection of a specific test depends on the PTP of stenotic CAD and other patient characteristics (e.g. presence of ECG or echocardiographic abnormalities already at rest and/or the inability to exercise), and local availability and expertise. The result of the non-invasive test will modify a subject’s PTP of stenotic CAD, and the resulting post-test probability will indicate whether or not ICA is indicated.

However, data showing a low yield of ICA [5] suggest that such an assessment is not always performed in clinical practice. Specifically, a relatively recent study from Switzerland claimed that more than one third of patients undergoing purely diagnostic ICA did not have any non-invasive test beforehand [6]. This study suggesting a significant overuse of ICA in Switzerland has led to intense discussions about the appropriateness of ICA and speculations about monetary interests of invasive cardiologist in performing expensive procedures in patients without a medical indication [7]. A closer look at this study however reveals that the underlying data are derived from insurance claims rather than a detailed review of patient histories [6], and that the lack of relevant information on the included patients (e.g. symptoms, risk factors) precludes definite conclusions about the appropriateness of ICA in Switzerland.

Therefore, we aimed to re-evaluate the practice of non-invasive testing prior to ICA in Switzerland by a systematic and detailed manual case-by-case review of consecutive patients undergoing ICA in a large referral centre in Eastern Switzerland during a period of one year.

## Methods

### Study design

This is a fully retrospective analysis of data which had been collected systematically for clinical purposes. Consecutive patients fulfilling the inclusion and exclusion criteria were identified using the ICA database of the cardiology department of the Kantonsspital St. Gallen and the planning tool (i.e. “Terminplaner”) manually on a case-by-case basis. The data required for the analysis were obtained from the clinic information system and the paper versions of the patient histories. All data were extracted manually by one single researcher. The data were fully anonymized before analysis. The study was approved by the ethics committee of the Kanton St. Gallen. The ethics committee waived the requirement for informed consent.

### Study population

The Kantonsspital St. Gallen is the main health care centre in Eastern Switzerland. In the cardiology department, which serves a population of approximately 700 000 inhabitants, approximately 2500 ICA per year are performed. We studied all patients >18 years undergoing elective ICA solely for the evaluation of stenotic CAD during the year 2015. Thus, patients with acute coronary syndrome were excluded. In addition, patients undergoing ICA in a specific context such as valve disease, heart failure and pulmonary hypertension were excluded. Patients undergoing scheduled interventions based on a previous ICA were also excluded.

### Assessment of pre-test probability

The PTP of stenotic CAD was determined by the revised Diamond-Forrester [8] model using an online calculator [9]. The PTP was calculated according to the “basic model” (type of symptoms, gender and age) and the “extended model” (plus cardiovascular risk factors smoking, hypertension, dyslipidemia, diabetes).

Typical chest pain was defined as having (i) substernal chest pain or discomfort, that is (ii) provoked by exertion or emotional stress and (iii) relieved by rest and/or nitroglycerine. Atypical chest pain was defined as having only two of the before-mentioned three criteria. If only one or none of the criteria was present, the patient was classified as having non-specific chest pain.

### Definition of non-invasive tests

In accordance with current guidelines [4] the following tests were considered as non-invasive tests: exercise stress test, stress echocardiography, myocardial perfusion scintigraphy, cardiac stress magnetic resonance, positron emission tomography, or computed tomography coronary angiography. The exercise stress tests were typically performed on full medication, i.e., antiischemic drugs were not stopped prior to the tests. Resting ECG and echocardiography (without stress) were not counted as non-invasive tests. For the present study we considered non-invasive tests performed within 6 months prior to ICA.

### Invasive coronary angiography

ICA was performed by femoral or radial approach using standard techniques. The severity of coronary stenosis as described by % lumen diameter reduction was assessed visually. Stenotic CAD was defined as any stenosis >50%. Patients with any lesion ≤50% were labelled as patients with “atherosclerosis”. Only patients with angiographically absolutely normal coronary arteries were diagnosed as having “normal coronary arteries”. The decision about revascularization and the mode of revascularization were at the discretion of the invasive cardiologist. This decision usually took into account the full clinical information, coronary anatomy, and also patient preference. In patients with ambiguous severity of coronary stenosis fractional flow reserve (FFR) is routinely measured at our institution as a basis to decide about revascularization.

### Statistical analysis

Patient characteristics are presented as numbers (percentages), mean ± standard deviation or median (interquartile range) as appropriate. The proportion of patients undergoing a non-invasive test prior to ICA was assessed in the entire population, in patients undergoing only diagnostic ICA (i.e., not followed by percutaneous coronary intervention or bypass surgery), and in patients with an intermediate PTP between 15 and 85%, i.e. the PTP range where non-invasive tests are recommended by guidelines. Patients undergoing a non-invasive assessment prior to ICA and those not doing so were compared using chi-square test, unpaired t-tests or Mann-Whitney-U-tests as appropriate. Independent predictors of the lack of a non-invasive test prior to ICA were assessed using multivariate logistic regression (backward stepwise model). A p value less than 0.05 was considered statistically significant. Statistical analysis was performed using SPSS version 20.0 (SPSS Inc, Chicago, Illinois).

## Results

### Study population

The entire study population consisted of 816 patients. The mean age was 67±9 years, and 70% were males. Patients had a typical risk factor profile (51% smoking, 88% with dyslipidemia, 75% with hypertension, and 22% with diabetes, and also they had a typical medication profile (Table 1). The median PTPs according to the basic and extended model were 37% and 44% respectively. The proportion of patients falling in the intermediate PTP range of 15–85% was 664/861 (81%) according to the basic model and 661/861 (81%) according to the extended model.

**Table 1:** Characteristics of the entire study population and patients undergoing a non-invasive test prior to invasive coronary angiography versus those not undergoing a test.

	All (n = 816)	Test (n = 764)	No Test (n = 52)	P value
Age (years)	67±9	67±9	69±10	0.08
Sex (male)	568 (70%)	535	33	0.32
Body mass index (kg/m^2^)	28.2±4.8	28.2±4.8	29.4±4.7	0.09
Cardiovascular risk factors				
Smoking	413 (51%)	384 (50%)	29 (56%)	0.44
Cholesterol	719 (88%)	673 (88%)	46 (88%)	0.94
Hypertension	608 (75%)	566 (74%)	42 (81%)	0.28
Diabetes	179 (22%)	164 (21%)	15 (29%)	0.21
Family history	310 (38%)	289 (38%)	21 (40%)	0.71
History of coronary artery disease	270 (33%)	250 (33%)	20 (38%)	0.40
Extracardiac atherosclerotic disease	138 (17%)	132 (17%)	6 (12%)	0.29
**Medication**				
Aspirin	635 (78%)	600 (79%)	35 (67%)	0.06
Oral anticoagulation	93 (11%)	82 (11%)	11 (21%)	0.02
Beta-blocker	469 (57%)	437 (57%)	32 (62%)	0.54
ACEI/ARB	440 (54%)	412 (54%)	28 (54%)	0.99
Other antianginal drug(s)	194 (24%)	175 (23%)	19 (37%)	0.03
**Symptoms**				0.99
Non-specific chest	175 (21%)	164 (21%)	11 (21%)	
Atypical angina	215 (26%)	202 (26%)	13 (25%)	
Typical angina	426 (52%)	399 (52%)	27 (52%)	
**Pre-test probability**				
Basic model	37 (19–61)	37 (19–61)	41 (16–62)	0.64
Basic model + risk factors	44 (22–67)	44 (23–67)	51 (19–71)	0.41
**Echocardiography**	765 (92%)	721	44	0.44
LVEF (%)	58±10	59±9	54±14	0.003

Data are given as numbers and percentages, mean±standard deviation, and median (interquartile range) as appropriate.

ACEI: angiotensin converting enzyme inhibitor, ARB: angiotensin receptor blocker, LVEF: left ventricular ejection fraction

### Non-invasive tests

As shown in Table 2, 764/816 patients (94%) had at least one non-invasive test prior to ICA. This was an exercise stress test in the vast majority of patients (89%). One fifth (160/816; 20%) of patients underwent a test other than an exercise stress test (6% scintigraphy, 4% stress echocardiography, 6% stress MRI, 4% computed tomography coronary angiography), and 122/816 (15%) patients had two test (exercise stress test and another test) (Table 2).

**Table 2:** Non-invasive tests in the entire study population (n = 816).

**Any test**	764 (94%)
Exercise stress test	728 (89%)
Work rate (Watt)	132±53
Rate pressure product (mmHg*min^-1^)*	25093±6709
≥25000 mmHg*min^-1^	337 (46%)
Rate pressure product factor*	2.6±0.8
≥2.5	349 (48%)
Abnormal test result (symptoms and/or ECG)	543 (75%)
Myocardial perfusion scintigraphy	48 (6%)
Abnormal test result	44 (92%)
Stress echocardiography	30 (4%)
Abnormal test result	22 (73%)
Stress MRI	47 (6%)
Abnormal test result	43 (92%)
Computed tomography coronary angiography	35 (4%)
Abnormal test result	34 (97%)
**More than one test**	
Exercise stress test + myocardial perfusion scintigraphy	36 (4%)
Exercise stress test + stress echocardiography	25 (3%)
Exercise stress test + stress MRI	31 (4%)
Exercise stress test + computed tomography	30 (4%)

Data are given as numbers and percentages, mean±standard deviation, and median (interquartile range) as appropriate.

ECG: electrocardiogram, MRI: magnetic resonance imaging.

*parameters considered to be indicative of a meaningful test

When looking only at patients with an intermediate PTP, the proportion of patients undergoing a non-invasive test was also 94% independently whether the PTP was calculated according to the basic (624/664) or extended (624/661) model. When looking only at patients with a purely diagnostic angiogram (i.e. not followed by any form of revascularization), the proportion of patients undergoing a non-invasive test was 346/374 (93%).

### Predictors of the lack of a non-invasive test

Patients undergoing a non-invasive test and those not doing so are compared in Table 1. It becomes obvious that the groups were very similar. In particular, the groups did not differ with respect to sex, risk factors, and PTP. The only differences between the groups were a higher proportion of patients on oral anticoagulation and on antianginal drugs other than beta-blockers, a lower left ventricular ejection fraction (LVEF) and trend towards higher age in the group of patients not undergoing a non-invasive test. Multivariate analysis revealed that the use of antianginal drugs other than beta-blockers [odds ratio 1.92 (95% confidence interval 1.01–3.66); p = 0.047] and lower LVEF [odds ratio 0.97 (95% confidence interval 0.94–0.99) per one % point increase in LVEF (p = 0.005) indicating a 3% lower likelihood of a non-invasive test with each % point increase in LVEF] were the only independent predictors for the lack of an invasive test

### Results of ICA

Stenotic CAD was found in 72% of patients, 24% had coronary atherosclerosis, and 4% had normal coronary arteries. Patients with and without non-invasive tests did not differ with respect to the findings of ICA (Table 3). Among all 816 patients, 442 (54%) patients underwent revascularization (percutaneous coronary intervention or coronary artery bypass grafting, Table 3). There was not difference in the management between patients with and without non-invasive tests either (Table 3).

**Table 3:** Findings from invasive coronary angiography and management in the entire study population and in patients undergoing a non-invasive test prior to invasive coronary angiography versus those not undergoing a test.

	All (n = 816)	Test (n = 764)	No Test (n = 52)	P value
**Coronary angiography**				0.68
Stenotic CAD (stenosis >50%)	584 (72%)	545 (71%)	39 (75%)	
atherosclerosis	195 (24%)	185 (24%)	10 (19%)	
Normal coronary arteries	37 (4%)	34 (4%)	3 (6%)	
** CAD: number of vessels**				0.38
1	143 (24%)	130 (24%)	13 (33%)	
2	176 (30%)	168 (31%)	8 (21%)	
3	265 (46%)	247 (45%)	18 (46%)	
**Treatment**				0.59
None	21 (3%)	20 (3%)	1 (2%)	
Medical therapy	353 (43%)	326 (43%)	27 (52%)	
Percutaneous coronary intervention	312 (38%)	294 (38%)	18 (35%)	
Coronary artery bypass grafting	130 (16%)	124 (16%)	6 (11%)	

Data are given as numbers and percentages.

CAD: coronary artery disease.

### Patients with stenotic CAD not undergoing revascularization

There were 146 patients (18% of the entire population) who fulfilled the criterion for “stenotic CAD” but did not undergo revascularization. In 34 of these patients, FFR measurements were performed in 45 vessels (mainly left anterior descending artery; mean FFR 0.91±0.05) as basis for a decision not to perform revascularization. Other reasons not to perform revascularization included visual grading of a stenosis as within the range between 50 and 70% by the invasive cardiologist (n = 42), lesions in small vessels with an unfavorable risk-benefit ratio for percutaneous coronary intervention (typically ostial diagonal branch lesions) (n = 31), complex anatomy but options for conservative therapy (n = 28), post-coronary artery bypass patients with patent grafts but diseased peripheral native vessels (n = 9), and an additional non-invasive test performed after ICA to exclude significant ischemia (n = 2).

## Discussion

In the present analysis of consecutive patients undergoing elective ICA in a large Swiss cardiology department during a period of one year, we showed that 94% of patients had undergone a non-invasive test prior to ICA. In addition, the yield of ICA was high with stenotic CAD in 72% of patients and subsequent revascularization in 54% of patients. These data point to an appropriate selection of patients for ICA based on PTP assessment, non-invasive testing, and clinical judgement. There is no evidence for an overuse of ICA.

The findings of our study are in clear contrast to the results of the study by Chmiel et al. [6] who have reported that one third of patients undergoing purely diagnostic ICA did not have any non-invasive test prior to ICA even though they also considered a resting echocardiogram as a non-invasive test although an echocardiogram at rest is not suited to provide information about inducible myocardial ischemia. Several aspects may account for the discrepant findings: first, a considerable number of patients nowadays undergo ICA in the context of valve disease. In these patients information on coronary anatomy is required to plan valve surgery/intervention [10], and ICA is typically performed based on the finding of severe valve disease requiring treatment rather than based on a non-invasive test pointing to inducible ischemia. Such patients may have been included in the study by Chmiel et al [6] but were excluded from the present analysis. Second, not all only diagnostic angiograms in the study by Chmiel et al. [6] may have been avoidable since ICA without subsequent revascularization is not always useless. Studies have shown that stenotic CAD can be managed medically in many patients, and that revascularization not always improves prognosis [11, 12]. However, to make a decision about conservative management or revascularization diagnostic ICA had been performed in all these studies [11, 12], and the same is necessary in daily practice. In line with this consideration 18% of the entire population of the present study had stenotic CAD defined as a stenosis > 50% in at least one vessel but did not undergo revascularization for a variety of good reasons as reported above. Third, a detailed analysis of the data by Chmiel et al. [6] is very difficult since hardly any patient characteristics (risk factors, symptoms, PTP) were reported. Thus, it is impossible to check the plausibility of the data and the detailed reasons leading to an angiogram. Although the authors claim the opposite, it remains possible that not all non-invasive tests in all patients were recorded. Fourth, Chmiel et al. [6] selected only patients undergoing ICA without subsequent revascularization while we included consecutive patients. However, when analyzing only this subgroup of patients not undergoing revascularization following ICA the proportion of patients undergoing a non-invasive test was similar as in the entire population (93 versus 94%).

Given these considerations we think that the study by Chmiel et al. [6] is not ideally suited to demonstrate that there is a general overuse of ICA in Switzerland. In contrast, our study showed that in the majority of patients the indication for ICA was based on the abnormal result of a non-invasive test. Although the exercise stress test has imperfect sensitivity and specificity and is recommended only for patients with a PTP in the lower two thirds of the intermediate range (15–65%) and patients not treated with antiischemic drugs [4], it was the main modality used in our population. Alternative tests allowing for localization and quantification of ischemia or non-invasive visualization of the coronary arteries were performed only in selected patients, most often in addition to an exercise stress. Still, this strategy obviously resulted in a high yield of stenotic CAD in those referred for ICA, presumably because factors other than the result of the exercise stress test such as history and findings from ECG and echocardiogram also contributed to the decision to perform ICA. In line with this, there was a substantial number of patients still referred for ICA despite a negative (or inconclusive) exercise stress test. Of course, the present study is not suited to determine in how many patients significant CAD had been missed due to the low sensitivity of the exercise stress test since this patients were not included in the study. Neither is the study suited to determine the accuracy of the non-invasive tests since we studied the selected population of patients referred for coronary angiography, most often based on positive test results and/or other indicators for stenotic CAD.

The high yield of stenotic CAD by ICA is seemingly in contrast to a large US study (nearly 400000 patients) by Patel et al. [5] who reported a prevalence of stenotic ICA of 38% in patients undergoing elective ICA with very similar inclusion and exclusion criteria as our study. These authors reported non-invasive testing in 84% of patients and a positive test result in 69% of all patients [5]. However, in this study a resting electrocardiogram and a resting echocardiogram were also considered as non-invasive tests, which is not in accordance with the current understanding of a non-invasive test for the evaluation of CAD [4]. This may explain the difference in CAD prevalence between the studies despite a seemly similar use of non-invasive tests and positive findings.

Some patients still underwent ICA without prior non-invasive testing. A comparison of patients undergoing a non-invasive test and those not doing so did not reveal major differences. One of the few differences was a lower LVEF in those without a test. Notably, guidelines suggest that in patients with reduced LVEF (less than 50%), and typical angina, ICA can be performed without additional non-invasive testing [4]. For such patients without documentation of the presence and localization of ischemia prior to ICA measurement of the FFR during ICA is nowadays an established and evidence based option to decide about revascularization [4, 12]. Additional differences were the higher proportion of antianginal drugs and a trend towards older age in those without a non-invasive test. In patients with PTP >85% (most often elderly men with typical angina) a diagnosis of CAD can be made without further non-invasive testing [4]. If such a patient is significantly limited by angina despite several antianginal drugs, ICA without further testing is an option. Measurement of FFR will then guide revascularization. Alternatively, non-invasive testing can be performed after diagnostic ICA to decide about revascularization in the presence of a complex coronary anatomy. Thus, the results of our multivariate analysis are plausible and further add to our interpretation of a reasonable selection of patients for ICA and a high adherence to guidelines during the evaluation of patients with suspected CAD in our area. Chmiel et al. [6] also tried to identify predictors for receiving or nor receiving a non-invasive test. They found that lower age and absence of antiplatelet therapy were associated with a lack of a non-invasive test. However, given the limited patient characteristics in that study, the information of this analysis is also limited.

A closer look at the PTP of our study population reveals that there must have been patients with a PTP less than 15%, i.e. patients in whom non-invasive testing is not recommended raising the question whether these patients really had an indication for ICA. In a recent analysis of computed tomography coronary angiography findings at our institution over a period of two years we have shown that PTP is imperfect for the prediction of stenotic CAD, and that also patients with PTP <15% may have stenotic CAD by computed tomography and ICA [13]. Thus, despite formally low PTP non-invasive testing and ICA may have been appropriate if clinical judgement raises the suspicion of CAD for reasons not reflected in the parameters determining PTP.

### Limitations

The present study has a number of limitations. First, it is a retrospective study. However, for the purpose of the present analysis this is not substantial limitation since all the required data could be collected in a retrospective manner. Second, the number of patients was relatively low, and the number of patients in the group without test prior to ICA was small limiting the power of the analysis on the predictors of the lack of a non-invasive test. However, the patient sample still allows a representative picture of the practice of testing prior to ICA, and the data quality was good. Third, our findings from Eastern Switzerland may not be representative for the entire country. Although some regional differences remain a possibility there is no reason to assume a significantly different practice of non-invasive testing prior to ICA in other parts of the country. When compared to Eastern Switzerland the density of cardiologist’s offices is much higher in other parts of the country (e.g. Zürich, Geneva, Basel, Berne), and therefore it is unlikely that less non-invasive tests are performed prior to ICA in these parts of the country. Fourth, since we only looked at patients undergoing ICA it remains unknown in how many patients stenotic CAD had been missed by the present approach. However, this was not the question of the study, but the study design is suited to address the criticism of an overuse of ICA derived from the study by Chmiel et al. [6].

## Conclusions

The present analysis of consecutive patients undergoing elective ICA in a large Swiss cardiology department during a period of one year showed that 94% of patients had undergone a non-invasive test prior to ICA, and that the yield of ICA was high with stenotic CAD in 72% of patients and consecutive revascularization in 54% of patients. Thus, patients for ICA are appropriately selected based on PTP assessment, non-invasive testing, and clinical judgement. There is no evidence for an overuse of ICA.

## Supporting information

S1 File Minimal data set. (XLSX)
